# Diffraction Methods for Qualitative and Quantitative Texture Analysis of Ferroelectric Ceramics †

**DOI:** 10.3390/ma14195633

**Published:** 2021-09-28

**Authors:** Chris M. Fancher

**Affiliations:** Materials Science & Technology Division, Oak Ridge National Laboratory, Oak Ridge, TN 37830, USA; fanchercm@ornl.gov

**Keywords:** ferroelectric, domain texture, grain texture, crystallographic texture, Rietveld, diffraction

## Abstract

Crystallographic textures are pervasive in ferroelectrics and underpin the functional properties of devices utilizing these materials because many macroscopic properties (e.g., piezoelectricity) require a non-random distribution of dipoles. Inducing a preferred grain texture has become a viable route to improve these functional properties. X-ray and neutron diffraction have become valuable tools to probe crystallographic textures. This paper presents an overview of qualitative and quantitative methods for assessing crystallographic textures in electroceramics (domain and grain textures) and discusses their strengths and weaknesses.

## 1. Introduction

Crystallographic textures are ubiquitous in materials. Materials have a texture or preferred orientation if the material has a non-random distribution of crystallite orientations. Textures arise because of the material process, e.g., forging [1,2], rolling [2] or additive [3] processing of metal and templated growth in bulk or thin-film ceramic processing [4] or because of material their use in service, e.g., mechanical and/or electrical loads [5,6]. The form and symmetry of the texture encode information about how the material is processed and subsequently utilized in applications [1,7]. Electroactive materials, such as ferroelectrics, derive their functionality from a non-random distribution of spontaneous dipoles [8,9,10].

Ceramic materials develop three preferred orientations; phase textures during transformation [11,12], ferroelastic domain texture [13,14,15,16], and grain texture [4,17]. The individual texture forms are not mutually exclusive for any specific sample, and thus a phase, domain, or crystallographic texture can coexist, resulting in a superposition of each texture. For example, electrical or mechanical stresses can induce a domain texture in ceramics with a crystallographic texture. The effect of the superposition of both crystallographic and domain texture was investigated in 00*l* oriented Bi_4.5_Na_0.5_Ti_4_O_15_ and 0.91Bi_1/2_Na_1/2_TiO_3_-0.07BaTiO_3_-0.02K_0.5_Na_0.5_NbO_3_ [18,19].

Quantifying the degree of induced orientation is essential when understanding the effects of processing or post-processing steps needed to achieve the desired material performance. X-ray and neutron diffraction techniques are valuable tools to determine the degree of orientation and the symmetry of the induced texture. In addition, diffraction techniques allow for direct pole figure measurement or reconstruction using Rietveld texture analysis. Advances in two-dimensional detectors [20,21] and time-of-flight neutron beamlines [22,23,24,25,26] enable the rapid collection of high-quality data for pole figure determination. These advances have also enabled direct observation of the evolution in domain textures using time-resolved scattering [27,28]. A variety of methods have been developed to extract qualitative (Lotgering or March–Dollase) or quantitative (probability densities) information from diffraction data [4,15]. This paper discusses the differences between qualitative and quantitative approaches for assessing crystallographic textures using X-ray and neutron diffraction.

## 2. Experimental Methods

### 2.1. Sample Preparation

Bulk Bi_1/2_Na_1/2_TiO_3_ and 0.91Bi_1/2_Na_1/2_TiO_3_-0.07BaTiO_3_-0.02K_0.5_Na_0.5_NbO_3_ (BNT-7BT-2KNN) ceramics were fabricated using conventional solid-state processing to produce randomly oriented materials or using the tape-casting method using a 5wt% BNT platelets for 00l oriented ceramics. Bi_1/2_Na_1/2_TiO_3_ (BNT) platelets with 00*l* perpendicular to their large face were prepared using a two-step molten salt method. See [29] for details about the seed processing and [30,31] for further information about ceramic processing.

### 2.2. Bragg-Brentano X-ray Diffraction

Conventional θ-2θ diffraction data were measured for textured and randomly oriented bulk ceramics using a Bruker D8 Focus (Bruker AXS, Madison, WI, USA). Diffraction data were measured using a 0.014° step size over a 20–80° 2θ scattering range. Integrated intensity for 7 sets of *hkl* reflections was extracted by single peak fitting using a Pseudo-Voigt function and linear background (*hkl* sets were fit over a narrow ±2θ range) using LMFIT [32].

### 2.3. Neutron Rocking Curves

Pole density measurements of textured BNT-BT-KNN were performed using neutron diffraction at Oak Ridge National Laboratory (ORNL) High Flux Isotope Reactor (HFIR) beamline HB3a [33]. After mounting, ω and χ scans were used to align samples relative to the 002 pole. The pole density of the 002 reflection was measured using an ω scan over a ±50° range from the aligned pole.

### 2.4. Angular Dependent Diffraction Data

Angular-dependent X-ray and neutron diffraction data were measured to compare the methods used for quantifying crystallographic texture using the Rietveld texture analysis approach. XRD data were measured using a Bruker AXS platform General Area Detector Diffraction System with a Hi-Star detector (Bruker AXS, Madison, WI, USA). The diffractometer used Cu Kα radiation with a graphite monochromator and 0.54 mm pinhole collimator. A sample was mounted in a two-stop fixed-chi Eulerian cradle, χ = 90° and 54°. Where χ denotes the rotation of the scattering vector from the out-of-plane (χ = 90°) toward the in-plane (χ = 0°). Data were measured during continuous rotation about the sample normal to improve counting statistics associated with large grains, imposing a fiber texture. Measured 2D XRD data were integrated using a 5° γ out-of-plane spacing (angular spacing along a diffraction arc). Neutron data were collected at the Australian Nuclear Science and Technology Organization Wombat beamline. The sample was mounted on a φ − χ Eulerian cradle to collect diffraction data every 5° in φ and 15° in χ. Wombat is equipped with a curved 2D position-sensitive detector covering 120° 2θ and 15° χ coverage. Each χ and φ step, diffraction data are binned into three χ sets (χ ± 5°) with a 0.125° 2θ increment. The resulting group of measured diffraction data sampled the pole figure with a grid spacing of 5° × 5° (1387 independent diffraction spectra).

The crystallographic texture of the BNT-BT-KNN was determined by Rietveld texture analysis (RTA) using the software package “materials analysis using diffraction” (MAUD) version 2.94 [34,35,36]. RTA was performed using discrete methods (E-WIMV or WIMV with a 5° resolution), and spherical harmonic methods were used to determine the ODF for the given diffraction data set [37,38]. XRD data were used to assess harmonic order cutoff (4 <= L_max_ <= 22) on the determined crystallographic texture. Neutron data were analyzed without an imposed sample symmetry to highlight the advantages of modeling textures using an ODF.

### 2.5. Field Dependent Diffraction

Electric field-dependent diffraction data were measured in situ at beamline 11-ID-C at the Advanced Photon Source (APS) of Argonne National Laboratory using 111 keV (0.11165 Å) X-rays. Diffraction images (2D data) were measured using a Perkin-Elmer 2D detector (Perkin-Elmer, Waltham, MA, USA) (2048 × 2048 pixels) positioned ~2.8 m from the sample. Measured data were corrected for detector tilt, beam center shift detector distance, then integrated into 36 slices (10°χ spacing) using the fast azimuthal integration using the python software package (pyFAI) [39]. The integrated intensity 111 and $1\bar{1}1$ reflections were extracted by single peak fitting using a pseudo-Voigt with a linear background using LMFIT.

## 3. Mathematical Methods

### 3.1. Lotgering Factor

A simple method, the Lotgering factor, to quantify the degree of orientation of a sintered ceramic is to compare the relative peak intensities of powder XRD spectrum [40]. Using the assumption that changes of the relative intensity of Bragg peaks are the result of a preferred grain alignment, the fraction of grains aligned along a specific direction can be estimated. The surface normal is the reference axis, but any direction can be used in principle. In BNT-based materials, the Lotgering factor compares the change in relative intensities of the 00*l* poles. The Lotgering method calculates a qualitative metric representing the texture of the sample using the following expression:

(1) $$f=\frac{p-p_{o}}{1-p_{o}};p=\frac{\sum^{\;}I_{00l}}{\sum^{\;}I_{hkl}};p_{o}=\frac{\sum^{\;}I_{00l}^{o}}{\sum^{\;}I_{hkl}^{o}}$$

where *I_hkl_* is the integrated intensity of the *hkl* pole, *p_o_* and *p* represent the fraction of the 00*l* poles for randomly oriented and templated samples, respectively. A θ-2θ scan is required to determine the Lotgering factor for a given sample accurately. Measuring the fraction aligned along the off-axis would require an uncoupled θ-2θ scan or a coupled θ-2θ scan with a χ-tilt. As the sample normal is the primary reference axis, the Lotgering technique would not capture the complex orientation symmetries associated with many deformation textures [1,22,23,41,42] and naturally occurring textures in biominerals [43,44].

### 3.2. March-Dollase

A more complete functional method to estimate the degree of orientation, proposed by March [45] and expanded by Dollase [46], describes the orientation of crystallites using a single function. The March–Dollase function uses two variables to describe the pole density using the following trigonometric function:

(2) $$P\left(r,\;\alpha \right)={\left(r^{2}\times \cos^{2}\;\alpha +\frac{1}{2}\times \sin^{2}\;\alpha \right)}^{-3/2}$$

where *r* is a constant that describes the degree of orientation and α is an arbitrary angle centered on the specific pole of interest. *P*(*r*,*α*) represents the probability of finding crystallites aligned at α off the pole of interest in units of multiples of a random distribution (MRD). The maximum texture for the pole of interest is at α = 0°, with a texture determined by:

(3) $${\left(r^{2}\times \cos^{2}\;0+\frac{1}{2}\times \sin^{2}\;0\right)}^{-3/2}={\left(r^{2}\right)}^{-\frac{3}{2}}=r^{-3}$$

The initial formulation by March (Equation (2)) assumes all crystallites have some degree of preferred orientation. Dollase modified Equation (2) to account for the case where a sample comprised of both a textured and randomly oriented volume fraction, defined as:

(4) $$P\left(r,\;\alpha ,\;x\right)=\left(1-x\right)+x\times {\left(r^{2}\times \cos^{2}\;\alpha +\frac{1}{2}\times \sin^{2}\;\alpha \right)}^{-3/2}$$

where *x* denotes the volume fraction of grains with a crystallographic texture, the fraction of textured grains in Equation (4) does not necessarily equal the fraction determined using the Lotgering method. The March–Dollase fit requires an axisymmetric texture symmetry such as the texture that develops from templated grain growth and reactive templated grain growth from isotropic templates [47].

### 3.3. Orientation Distribution Functions

The orientation of any crystallite, with respect to an external reference frame, can be described using a set of three rotation angles represented as $\left(\phi_{1},\;\Phi ,\;\phi_{2}\right)$ using Bunge notation. Where $\phi_{1,2}$ and Φ are rotation angles about z, x’, and x, respectively. The orientation distribution function (ODF) represents the probability of a crystallite having a specific set of $\left(\phi_{1},\;\Phi ,\;\phi_{2}\right)$ angles. Evaluating the ODF for one particular $\left(\phi_{1},\;\Phi ,\;\phi_{2}\right)$ gives the ratio of a crystallite having a specific orientation compared to a randomly oriented sample. ODFs allow for the reconstruction of inverse pole figures and pole figures for any arbitrary pole.

Conventional diffraction techniques only provide information about two Euler angles. As a result, multiple methods have been developed to reconstruct the full ODF from the incomplete set of crystallite orientations. Two approaches have become widespread: the spherical harmonic method developed by Bunge [48] and Roe [49,50], and the discrete method developed by Matthies and Vinel [51]. Unlike both the Lotgering and March–Dollase methods, ODF methods allow for determining a probability distribution function representing all orientation space. Given a complete or incomplete set of pole figure measurements, pole figure inversion can reconstruct the ODF. Mathematically, ODF models of $f\left(\phi_{1},\;\Phi ,\;\phi_{2}\right)$ are a probability distribution describing the distribution of crystallites in orientation space. ODF models determine the ODF through an inversion of the following:

(5) $$P_{hkl}\left(\alpha ,\;\beta \right)=\frac{1}{2\pi }\int_{0}^{2\pi }f\left(\phi_{1},\;\Phi ,\;\phi_{2}\right)d\Gamma$$

where α and *β* are the polar and azimuth angle of the *hkl* pole figure, and (*φ*_1_,*Φ*,*φ*_2_) are the Bunge Euler angles. Thus, a pole figure inversion of a set of known poles, *P_hkl_*(*α*, *β*), allows the determination of a pole figure for an unknown pole. In recent years, ODF models have been incorporated into Rietveld refinement programs.

#### 3.3.1. Harmonic ODF

Bunge [48] and Roe [49,50] independently developed similar harmonic methods. Both are practically similar, but the formulation of each is slightly different. Therefore, only Bunge’s is reviewed in this section. See [2] for an overview of Roe’s method. Bunge expands Equation (5) in terms of spherical harmonics:

(6) $$P\left(r,\;\alpha \right)=\overset{\infty }{\underset{l=0}{\sum }}\overset{N\left(l\right)}{\underset{\nu =1}{\sum }}F_{l}^{\nu }{\dot{k}}_{l}^{n}\left(\alpha ,\;\beta \right)$$

where $F_{l}^{\nu }$ are the pole figure coefficients and ${\dot{k}}_{l}^{n}\left(\alpha ,\;\beta \right)$ are symmetrized spherical harmonics. The orthogonality of spherical harmonics allows for a pole figure’s coefficients to be determined by integrating experimental pole figures.

(7) $$F_{l}^{\nu }=\int_{0}^{\pi }\int_{0}^{2\pi }p\left(\alpha ,\;\beta \right){\dot{k}}_{l}^{n}\left(\alpha ,\;\beta \right)sin\alpha d\beta d\alpha \;$$

The Bunge method expands the ODF using a series of generalized spherical harmonic functions:

(8) $$f\left(\phi_{1},\Phi ,\;\phi_{2}\right)=\overset{\infty }{\underset{l=0}{\sum }}\overset{M\left(l\right)}{\underset{\mu =1}{\sum }}\overset{N\left(l\right)}{\underset{\nu =1}{\sum }}C_{l}^{\mu \nu }{\ddot{T}}_{l}^{\mu \nu }\left(\phi_{1},\;\Phi ,\;\phi_{2}\right)\;$$

where $C_{l}^{\mu \nu }$ are coefficients of the ODF that are determined by fitting experimental pole figures and ${\ddot{T}}_{l}^{\mu \nu }\left(\phi_{1},\;\Phi ,\;\phi_{2}\right)$ are the generalized spherical harmonic functions.

Errors associated with a finite harmonic cutoff introduces one of the main limitations of the harmonic method. Materials with strong textures magnify these errors because large regions of orientation have a zero or near-zero $f\left(\phi_{1},\;\Phi ,\;\phi_{2}\right)$ [2,48]. Spherical harmonics of even and odd harmonic orders are required to have symmetric and antisymmetric symmetry, respectively. Friedel’s law requires diffraction to have a centrosymmetric symmetry, requires all odd order $F_{l}^{\nu }$ to equal zero. The harmonic ODF represents a summation of odd and even components:

(9) $$f\left(\phi_{1},\Phi ,\;\phi_{2}\right)=\tilde{f}+\tilde{\tilde{f}}$$

where $\tilde{f}$ and $\tilde{\tilde{f}}$ are the even and odd components, respectively [2]. X-ray and Neutron techniques can only directly determine even components. While all methods are susceptible to false or ghost peaks, the spherical harmonics do not have an implicit method to account for ghost peaks. Another limitation to the spherical harmonics arises because the represented ODF has a finite cutoff harmonic order. As a result, pole figures reconstructed using the spherical harmonic method can contain negative ODF values. Vanhoutte proposed the exponential harmonic as an extension to account for ghost corrections and ODF positivity [52].

#### 3.3.2. Discrete ODF

The discrete method has evolved since first proposed by Williams in 1968 [53]. Later additions by Imhof [54] then Matthies and Vinel [51] resulted in a model that took the names of the contributors Williams–Imhof–Matthies–Vinel (WIMV). Discrete methods treat the ODF as a set of discrete bins with a specific angular size. Unlike the harmonic method, matrix inversion of the following expression is used to solve the ODF:

(10) $$P_{hkl}\left(\alpha ,\;\beta \right)=\frac{1}{N}\overset{N}{\underset{i=1}{\sum }}f\left(\left(\alpha ,\;\beta \right)⇐{\;(\phi_{1},\Phi ,\;\phi_{2})}_{i}\right)$$

the summation is performed over orientation cells that contribute to the final cell (α,β). The WIMV method determines an initial guess for solving the resulting set of linear equations through a determination of the geometric mean of the experimentally measured pole figure, mathematically represented as:

(11) $$f_{o}\left(\phi_{1},\Phi ,\;\phi_{2}\right)=N_{o}\overset{I}{\underset{j=1}{\prod }}\overset{M_{i}}{\underset{m_{i}=1}{\prod }}p_{h_{i}}^{exp}{\left(\left(\alpha ,\beta \right)m_{i}\right)}^{\frac{1}{IM_{i}}}$$

where *I* is the number of experimental pole figures, *m_i_* is the multiplicity of the *i*-th pole, and *N_o_* is a normalization factor. Using a Rietveld analysis, the number of measured pole figures equals the number of poles in the refined spectra for a pole figure inversion. The final ODF is determined by minimizing the error between the calculated and experimental pole figures.

(12) $$f_{n+1}\left(\phi_{1},\Phi ,\;\phi_{2}\right)=N_{n}f_{n}\left(\phi_{1},\Phi ,\;\phi_{2}\right)\frac{f_{n}\left(\phi_{1},\Phi ,\;\phi_{2}\right)}{\prod_{j=1}^{I}\prod_{m_{i}=1}^{M_{i}}p_{h_{i}}^{n}{\left(\left(\alpha ,\beta \right)m_{i}\right)}^{\frac{1}{IM_{i}}}}$$

A method has been determined to help eliminate ghosts from appearing in a WIMV ODF. Allowing the refinement to impose an isotropic background such that:

(13) $$f\left(\phi_{1},\Phi ,\;\phi_{2}\right)\geq f_{\min}$$

suppress ghosts by eliminating negative holes corresponding to ghost peaks.

Similar to the harmonic method, more complex WIMV derivatives have been developed to improve the pole figure extraction, including the arbitrary defined cells (ADC) [55] and extended WIMV (EWIMV) [56]. Schaeben’s EWIMV method has been implemented in Rietveld packages using the following:

(14) $$f^{i+1}\left(\phi_{1},\Phi ,\;\phi_{2}\right)=f^{i}\left(\phi_{1},\Phi ,\;\phi_{2}\right)\overset{M_{hkl}}{\underset{m=1}{\sum }}{\left(\frac{P_{hkl}\left({\left(\phi_{1},\Phi ,\phi_{2}\right)}^{-1},h_{m}\right)}{P_{hkl}^{i}\left({\left(\phi_{1},\Phi ,\phi_{2}\right)}^{-1},h_{m}\right)}\right)}^{\lambda \frac{w_{hkl}}{M_{hkl}}}$$

where *M_hkl_* is the number of discretization points used for the integration of orientation space around pole *hkl*, *w_hkl_* weights the reflection to account for peak overlap, and $P_{hkl}^{i}({\left(\phi_{1},\Phi ,\phi_{2}\right)}^{-1},h_{m})$ is the calculated pole figure for the *i*-th iteration [44].

### 3.4. Dipole Distribution

Li et al. first reported that systematic changes in diffraction data are helpful to identify the signature of a change in the microscopic distribution of dipoles [57]. Changes in the distribution of spontaneous dipoles impact the intensity ratio of only non-degenerate reflections. In 2001, Wan and Bowman [58] proposed utilizing diffraction data to quantify the dipole orientation distribution (DOD). Jones et al. expanded the methods [59,60]. The development of DODs enabled the determination of the effect of electric and stress fields on the dipole evolution in ferroelectrics which provides insight into the functionality of these materials. Researchers can determine a DOD utilizing either a conventional ODF model in a Rietveld texture analysis or directly via single-peak fitting of measured diffraction. Single-peak fitting data are used to determine DODs using the following relationships for material with a rhombohedral long-range symmetry:

(15) $$f_{111}\left(\alpha \right)=4*\frac{\frac{I_{111}\left(\alpha \right)}{I_{111}^{\prime }}}{\frac{I_{111}\left(\alpha \right)}{I_{111}^{\prime }}+3\frac{I_{1\bar{1}1}\left(\alpha \right)}{I_{1\bar{1}1}^{\prime }}}$$

A DOD is commonly used to determine the fraction of dipoles oriented along a specific direction [60]:

(16) $$\eta_{111}\left(\alpha \right)=\frac{f_{111}\left(\alpha \right)}{4}-\frac{1}{4}=\frac{\frac{I_{111}\left(\alpha \right)}{I_{111}^{\prime }}}{\frac{I_{111}\left(\alpha \right)}{I_{111}^{\prime }}+3\frac{I_{1\bar{1}1}\left(\alpha \right)}{I_{1\bar{1}1}^{\prime }}}-\frac{1}{4}$$

where $I_{hkl}$ are the integrated intensities of the 111 and $1\bar{1}1$ reflections and $I_{hkl}^{\prime }$ are the integrated intensity of the hkl reflection with a random DOD (i.e., has not experienced mechanical or electric fields). Note that the $1\bar{1}1$ represents the combination of the $\bar{1}11$, $1\bar{1}1$, and $11\bar{1}$ reflections. Obtaining correct intensities for a random DOD state is essential to constrain the normalization. These data are routinely obtained from the material before being subjected to a field that would bias the distribution of dipoles. Alternatively, the random state can be inferred from angular dependent diffraction data using:

(17) $$I_{hkl}^{\prime }=\int_{\alpha =0}^{90}I_{hkl}\left(\alpha \right)\sin\left(\alpha \right)d\alpha$$

Diffraction data are measured at discrete intervals and not measured over a continuous set of angles. Therefore, Equation (17) is discretized as:

(18) $$I_{hkl}^{\prime }=\overset{90}{\underset{\alpha =0}{\sum }}I_{hkl}\left(\alpha \right)\left[\cos\left(\alpha -\frac{\Delta \alpha }{2}\right)-\cos\left(\alpha +\frac{\Delta \alpha }{2}\right)\right]$$

where Δα represents the out-of-plane grid spacing or angular integration range used to reduce 2D data into separate 1D patterns. The summation is discontinuous at the boundary conditions (α=0 and α=90), where $\left[\cos\left(\alpha -\frac{\Delta \alpha }{2}\right)-\cos\left(\alpha +\frac{\Delta \alpha }{2}\right)\right]$ is 0. Instead, these boundaries are treated as 2$\times \left[1-\cos\left(\frac{\Delta \alpha }{2}\right)\right]$ and 2$\times \left[\cos\left(90-\frac{\Delta \alpha }{2}\right)\right]$, respectively.

## 4. Results and Discussion

### 4.1. Lotgering Factor

**Figure 1:**
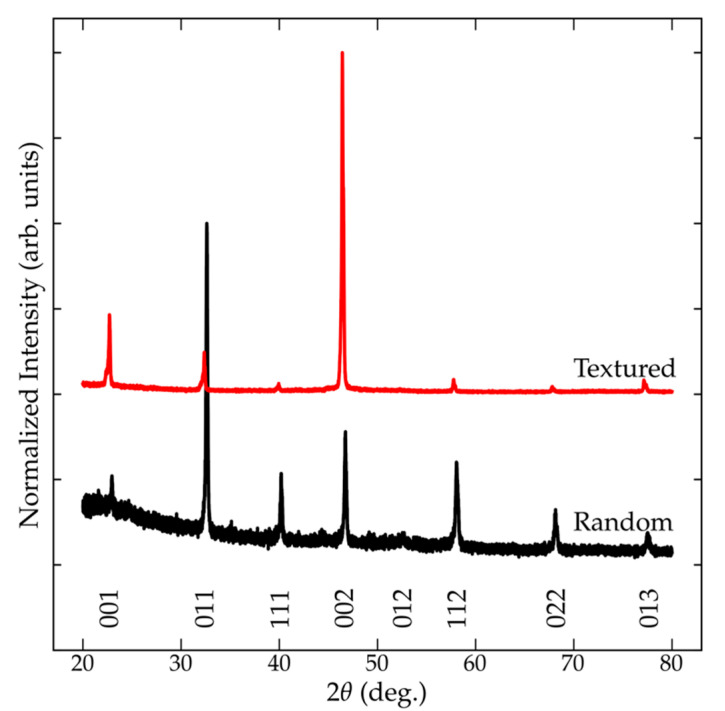
Comparison of the θ-2θ diffraction patterns for a randomly oriented and textured BNT-BT-KNN ceramic.

The Lotgering factor is determined based on the intensities measured using a θ-2θ pattern. Figure 1 compares the XRD patterns for randomly oriented and textured BNT. The measured θ-2θ diffraction data of textured BNT shows the characteristics of a strong 00*l* preferred orientation, where the 00*l* reflections account for much of the measured diffraction signal. Unlike a randomly oriented ceramic, the diffraction signal from templated material shows the strongest reflection from the 00*l*. The pseudocubic structure allows for all reflections to be treated as a single reflection. See Table S1 for a summary of the extract peak intensities.

The integrated intensity of each *hkl* within the 20° to 80° range was used to determine the relative fraction of the 001 contributions to the total scattered intensity (*p* or *p_o_* in Equation (1)). A *p* and *p_o_* of 0.845 (3) and 0.193 (3) were calculated for the textured and randomly oriented BNT ceramics. The high *p* for the textured sample is indicative that 00*l* dominantly contributed to the measured diffraction data. The textured ceramic was found to have a high f of 0.809 (2). A Lotgering factor for ideal *p_o_*-value for a theoretical pattern for the crystallographic structure of BNT was also determined. Table 1 compares the Lotgering factors determined for an experimental and predicted reference. Even though the *p_o_* for the predicted pattern is slightly lower (0.188 compared with 0.193 (3)), the resulting Lotgering factors are similar and within the estimated uncertainty. It should be noted that this indicator only considers a single set of diffraction data and does not provide a metric to determine crystallographic distributions within a given sample.

### 4.2. March–Dollase

The March–Dollase formulation adds additional complexity over the Lotgering method by incorporating angular-dependent diffraction information to gauge crystallographic texture based on systematic changes in the intensity of diffraction X-rays or neutrons. March–Dollase parameters are determined using Rietveld refinement [46] or fitting Equations (2) or (3) to pole density measurements [61,62]. The latter approach is discussed here for simplicity. Pole densities are measured in reflection geometry using either an ω-scan and χ-scan using an area detector or four-circle goniometer. ω-scans are performed by measuring the diffraction signal with the detector fixed at the Bragg angle while moving the incident angle. In contrast, χ-scans fix θ and 2θ to the Bragg reflection and pole density information is collected by varying the sample tilt (four circle goniometer) or integrating along the measured diffraction ring.

**Figure 2:**
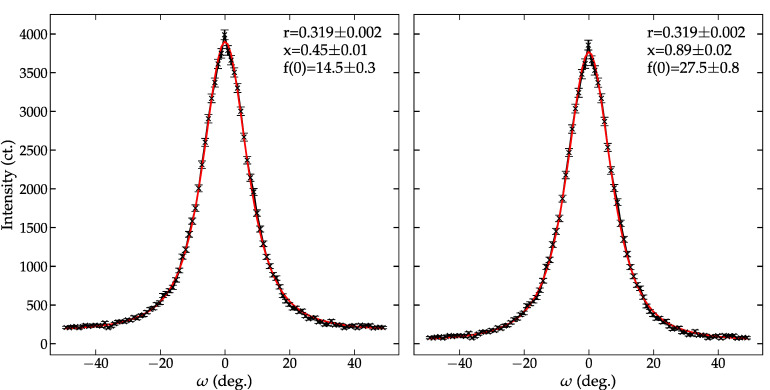
Measured 200 pole density of BNT-BT-KNN were of as measured (**left**) and background subtracted (**right**) BNT-BT-KNN (X) overlaid with a March–Dollase functional fit.

Measuring pole densities with an x-ray source requires non-linear absorption correction accurate r and x [62]. Uncoupled θ-2θ scans of the Bragg reflection are one route to correct the measured pole density for non-linear absorption [56]. Pole density measurements using an area detector or four-circle goniometer offer an advantage to a ω-scan because of the allowed angular range. The geometry of ω-scans limits the available orientation sampling to a ±θ_B_ angular range because the sample blocks the diffracted X-rays (θ_B_ > α) or incident X-rays are blocked (θ_B_ < α). In principle, diffraction collected using an area detector or four-circle goniometer would allow an angular range of ±~80° before the sample would block the diffraction. Materials with a low-angle texture pole benefit from the low-angle orientation permitted range with an area detector. An alternative is to measure the pole density using neutron diffraction, where the low material absorption does not restrict the allowed angular range or require absorption corrections [2]. See Figure 2 for a comparison of a pole density before and after correcting for background contributions. 

The measured 200 pole density of BNT-BT-KNN was analyzed using the March–Dollase formulation to estimate the crystallographic texture of the sample. Measured data were analyzed before and after accounting for background contribution to the measured data. Figure 2 compares the resulting pole densities and March–Dollase fits. Background subtraction does not impact the texture index (r) but substantially affects the fraction of textured material. The difference in the estimated volume fraction substantially increases the estimate of the maximum texture from 14.5 (3) to 27.0 (8) MRD. The full-width half max of the pole density plot (~17°) agrees with the March–Dollase fit predicting a strong texture. The increase in estimated texture highlights the importance of properly account for the scattering information used in the analysis. Uncorrected data would suggest a near equal mixture of random and oriented material. The subtraction of a constant background brings the fraction of templated material in line with the fraction of templated material (89 (2)%) predicted by the Lotgering method 80.7(2)%. The slight discrepancy could arise from various sources of error: (1) θ-2θ scans used only span up to 80° and introduce a bias in the p for the randomly oriented material and (2) the 200 pole density was measured using neutron diffraction, which probes the bulk instead of the top <20μm surface in XRD [63].

Note that the ω-scan used here only covers ± 45°. Neutron measurements do not suffer from the same absorption limitations observed in X-ray and can measure a complete ± 90° data set. These data were not utilized here because the traditional March–Dollase formulation is not suited to capture an increase in the 200 intensity observed at ω of 90°. A modified approach that takes accounts for *hkl* variants is needed to analyze a full ω-scan [64].

### 4.3. Rietveld Texture Analysis

Unlike the Lotgering and March–Dollase methods, ODF methods allow for determining a probability distribution function representing all orientation space. Thus, pole figure inversion methods can reconstruct the ODF given complete or incomplete pole figure measurements. Mathematically, ODF models of $f\left(\phi_{1},\;\Phi ,\;\phi_{2}\right)$ are a probability distribution describing the distribution of crystallites in orientation space. The harmonic [48,49,50] and discrete [65] methods have emerged as the primary ODF techniques. Pole figure Inversion is performed in Fourier (harmonics) or orientation (discrete) space. Each model has a different advantage and disadvantage.

**Figure 3:**
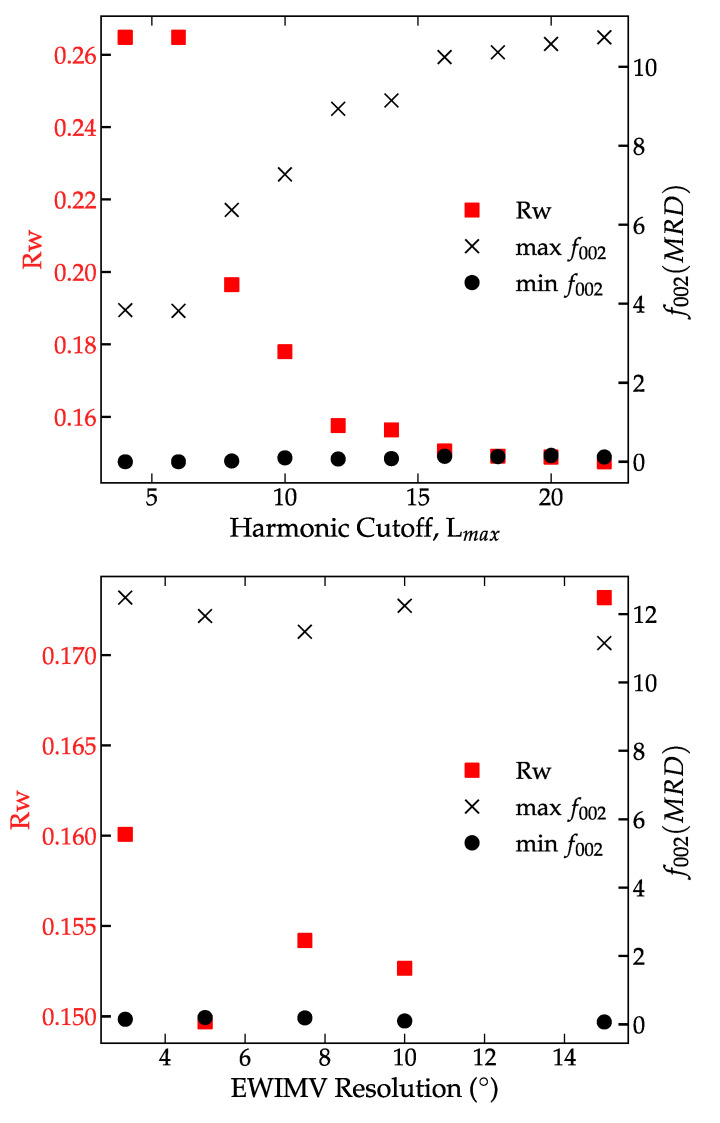
Comparison of the effect of spherical harmonic order (**top**) and EWIMV ODF resolution (**bottom**) on the texture and refinement error of BNT-BT-KNN sintered ceramics. Min and Max of f_002_, refinement error Rw are plotted against harmonic cutoff and EWIMV resolution.

**Figure 4:**
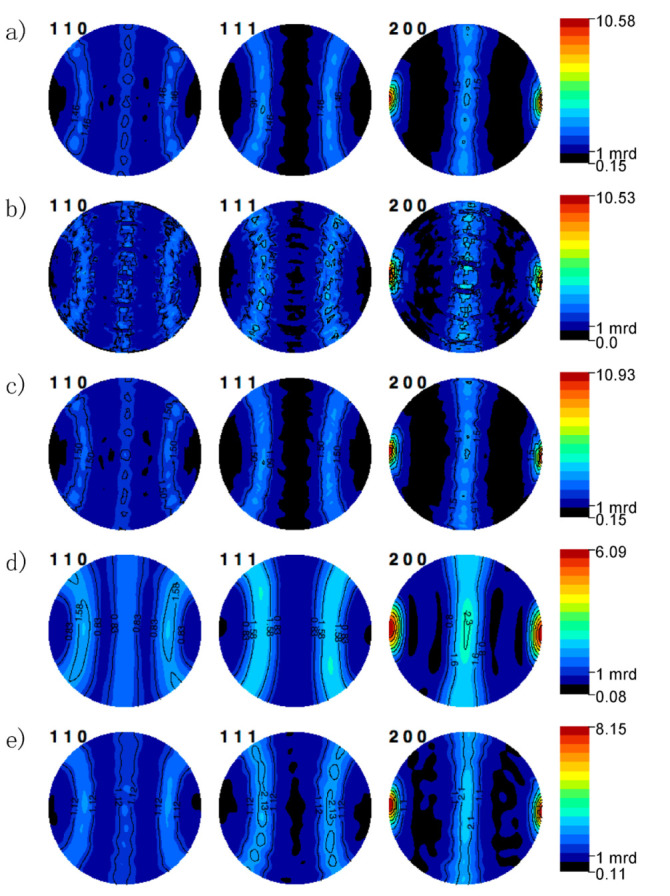
Comparison of the reconstructed pole figure refined using from neutron diffraction pole figure collected on a 5° spacing with (**a**) experimental reconstruction, (**b**) WIMV, (**c**) E-WIMV, (**d**) Spherical Harmonic *n* = 10, and (**e**) spherical harmonic *n* = 20. Note that the pole figures are rotated such that the left and right represent the sample normal direction.

The effect of harmonic cutoff order on the Rietveld results (texture and refinement errors) was explored using an imposed fiber texture for simplicity. Figure 3 the refined texture and Rietveld refinement error for the spherical harmonic refined using a harmonic order (L_max_) between 4 and 22, the maximum cutoff implemented in MAUD. Using a standard *n* = 4 harmonic cutoff, the resulting refined ODF has a negative texture and high refinement error (R > 20%). Increasing the harmonic cutoff above *n* = 10 removes the negative texture in the 00*l* ODF, and the resulting refinement error is significantly improved. Increasing harmonic cutoff above L_max_ = 4 has minimal impact on the final Rietveld refinement error, but the final refined texture increases linearly with harmonic cutoff. From L_max_ = 10–22, the weighted refinement error and the MRD increased. In contrast, the overall texture and Rietveld refinement errors are far less EWIVM resolution, see Figure 4. EWIMV refines a higher overall texture and achieves a similar refinement error as a high harmonic cutoff (L_max_ > 12). The EWIMV consistently determined a sharper overall texture. The apparent discrepancy between the harmonic and discrete methods could arise from the underlying differences in their formulation. The harmonic texture has implicit smoothing that can impact strong textures.

Texture refinement using no sample symmetry confirms the texture that develops from templated grain growth has an antisymmetric symmetry. A comparison of the reconstructed pole figures from different texture models to an experimental pole figure, reconstructed in Figure 4, shows the differences between each method and the ability of each technique to fit an experimental pole figure, as seen in Figure 4a. To directly compare the refined texture levels, each pole figure reconstruction and the experimental pole figure reconstruction have an associated color-coding unique to each method.

Reconstructed pole figures using the discrete method capture the overall texture profile and levels, Figure 4b,c. The standard WIMV method under-represented the minimum texture level and showed three circular oscillation artifacts are not present in the experimental reconstruction or recalculated pole figures using other methods. In addition, reconstructed pole figures show strong secularity that is not present in the reconstructed or EWIMV pole figures. EWIMV pole figure reconstructions closely reproduced the features of the experimental reconstruction, with the exception that the EWIMV method predicted a slightly stronger texture, 10.9 instead of 10.6. Spherical harmonic texture utilizing a harmonic cutoff of 10 and 20 are shown in (Figure 4d,e). These highlight errors that are introduced from a low harmonic cutoff. In addition to an increased refined maximum texture, subtle variations in the experimental pole figure missed with L_max_ = 10 are captured with a L_max_ = 20. Even with a high harmonic cutoff, the effect of Fourier smoothing is apparent. The refinement of an unconstrained harmonic texture model with a L_max_ = 20 required ~12 h to reach 5 convergence iterations. A harmonic texture analysis using a different Rietveld program, such as GSAS, might allow for a better-reconstructed pole figure fitting the experimental data because GSAS allows up L_max_ = 36. Both spherical harmonic pole figure reconstructions fit the experimental reconstruction better than the pole figure reconstruction using the exponential harmonic method.

Beyond differences in the resulting texture refinements, each texture model introduces different computational complexities. The Rietveld texture refinement required ~10 min for the discrete texture models (WIMV and EWIMV) to reach convergence, while the harmonic model converged after >12 h. These refinements utilized information from 1387 independent diffraction spectra. The WIMV and EWIMV methods refine the texture through a set of linear equations. The complexity of an unconstrained high-order harmonic model required significant computation time to reach a final pole figure inversion. Individual harmonic coefficients are determined through least-squares minimization in each Rietveld iteration. For example, a L_max_ of 10 and 20 without an imposed sample symmetry introduces 60 and 361 additional coefficients, introducing complexities for reaching convergence. Analyzing materials lower symmetry (e.g., orthorhombic, trigonal, or tetragonal) would further increase the number of coefficients.

### 4.4. Ferroelastic Domain Texture

**Figure 5:**
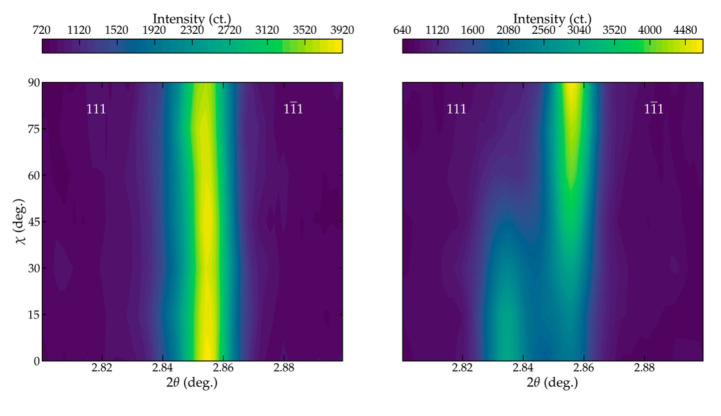
Measured angular dependent diffraction data for BNT before (**left**) and after (**right**) application of strong electric fields (8 kV/mm) for 1 s. Data are plotted vs. the out-of-plan angle χ, with 0 and 90 representing data measured parallel and perpendicular to the applied electric fields, respectively. Electric poling drives substantial ferroelastic domain wall motion with aligns the 111 lattice planes in the field direction (χ of 0).

Application of strong electric fields to ferroelectric ceramics biases the orientation of the spontaneous dipole into the direction of the electric field. The subsequent change in the directionality of the spontaneous dipoles underpins the functionality of ferroelectrics and leaves a signature in measured XRD data. Figure 5 compares the angular dependent data before (left) and after (right) application of electric fields. Application of a strong electric field (8 kV/mm) induces substantial ferroelastic domain wall motion, as evidenced by a significant increase in the intensity of the 111 reflections measured parallel to the applied electric field (χ = 0). The reorientation of dipoles into the electric field direction impacts the probability of finding a dipole with a given orientation or induces a texture. This effect is pronounced in Figure 5, where the intensity of the 111 peaks is maximized at χ = 0 and minimal at χ = 90.

**Figure 6:**
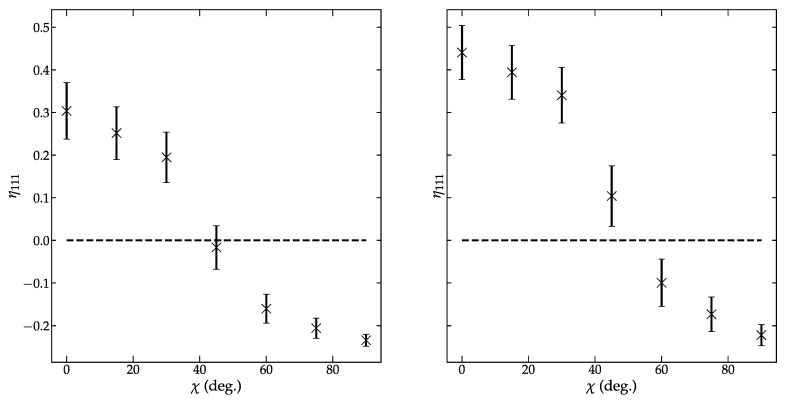
Normalization has a substantial impact on the determined fraction of domains aligned into the field direction. Data normalized using an experimental reference though peak fitting of data before poling (**left**) estimates a higher degree of domain alignment than a calculated equivalent random intensity (**right**).

Diffraction data measured before and after electric poling were analyzed by single peak fitting to extract information about the intensity of the 111 and $1\bar{1}1$ peaks. See Figures S1 and S2 for representative fits of the before and after data. Peak fit results were used to determine the extent of domain reorientation. The angular dependence of the fraction of aligned domains is shown in Figure 6. Note that a negative η_111_ indicates that the fraction of domains present is less than expected before electric poling. These data evidence that extensive domain reorientation has occurred with BNT achieving an η_111_ that suggests >40% of the available domains switched into the field direction.

The 111 reflections before electric poling showed minimal peak separation, making the peak fitting more complicated (see Figure 1). Data measured after the application of electric fields showed additional peak splitting with well-defined 111 and $1\bar{1}1$ peaks. The experimental reference resulted in a 111/$1\bar{1}1$ intensity ratio of 0.41, which is higher than the expected 1/3 ratio for a rhombohedral-like material. Equation (18) was used to estimate an equivalent random intensity for the 111 and $1\bar{1}1$ for comparison. The equivalent random intensity increased η_111_ compared to what was calculated using an experiment reference. While η_111_ increased from 0.30 (6) to 0.44 (6) when the equivalent reference was used, the reduction is still within the estimated error. Although the estimated 111/$1\bar{1}1$ intensity ratio of 0.23 is well below 1/3 and would overestimate η_111_. It is noted that the use of a coarse integration range (15°) can introduce numerical errors associated with the discretization of the integral (Equation (17)). The resulting error could account for the apparent discrepancy. Decreasing the integration range would help reduce the discretization error.

In addition to a direct DOD approach, researchers have also utilized ODFs to study the evolution in domain texture with applied electric fields. A Rietveld texture approach has the added benefits. It is well suited to decouple the texture in different phases and does not rely on an experimental reference for normalization [66]. These benefits make a Rietveld approach ideal for studying the evolution in domain alignment in materials prone to polymorphic phase transitions [19,67]. The precise formulation of Equations (15) and (16) changes slightly for different crystallographic symmetries [15]. Information about crystallographic symmetry is built into the ODF. Equation (15), and the variants for other symmetries, are often utilized in an axisymmetric formulation. Wang and Daniels utilized a Rietveld texture approach with and without an imposed fiber symmetry to determine that the commonly used fiber texture formulation is a good approximate. However, this assumption is invalid if the material has a crystallographic texture that breaks fiber symmetry or was subjected to a multi-axis load (e.g., cross poling) [68].

## 5. Comparison of Methods

Limitations of the Lotgering method prevent this method from being useful for more than an indication of the fraction of oriented grains. A lack of information from off-angle directions prevents an accurate determination of the distribution of templated grains. The March–Dollase function goes beyond the Lotgering method by including off-axis orientation information; however, the functional fit is only valid for materials over a select angular range in orientation space (e.g., ±54° although the range is dependent on the texture strength), and is restricted for use in materials with a fiber texture symmetry. At angles out of this range, the diffraction intensity increases because symmetry forces 00*l* variants to appear every 90°. The refined March–Dollase fit, r = 0.319 (2), over-estimates the MRD, 14.5. When a constant background is considered, the expected texture increased. Both estimates are higher than the texture level determined using Rietveld texture analysis or a direct method. Table 2 summarizes the advantages and limitations of the methods discussed in the paper

## Figures and Tables

**Table 1:** Summary of Lotgering factor for textured BNT-BT-KNN determined using either an experimental reference (powder diffraction pattern) or theoretical reference (modeled diffraction pattern from the crystallographic model). In addition, estimated errors in the Lotgering factor are reported in the parenthesis.

Lotgering Factor	Experimental Reference	Predicted Reference
f	0.945 (7)	0.946 (7)

**Table 2:** Summary of the benefits and limitations of the qualitative and quantitative methods for assessing crystallographic textures in ferroelectric ceramics.

Methods	Benefits	Limitations
Lotgering Factor	Qualitative metric to assess the volume fraction of textured material. Helpful in determining the effect of processing on the crystallographic texture.	Only utilizes a single diffraction pattern. Analysis requires data measured using a Bragg-Brentano geometry and does not provide information about angular dependences of the texture.
March–Dollase	Determines an estimate for the crystallographic texture of a given *hkl* pole of interest.	The commonly used formulation is limited to the analysis of materials with a fiber texture. Researchers must account for background and absorption contributions. Analysis requires angular-dependent diffraction data.
ODFs	Rigorous method for determining the strength and symmetry of a materials crystallographic texture.	Determining an ODF through Rietveld texture analysis requires information about the crystallographic structure (space group and atomic positions). Analysis requires angular-dependent diffraction data.
DOD	Metric to quantify the evolution in the domain alignment in response to a thermomechanical poling process.	Requires precise information about the initial state with a random domain configuration.

## Data Availability

The data presented in this study are available on request from the corresponding author.

## Supplementary Materials

The following are available online at https://www.mdpi.com/article/10.3390/ma14195633/s1, Table S1: Summary of the hkl integrated intensity used to determine the *p* for the templated and *p_o_* randomly oriented metric for an experimental (Powder) and simulated data (Structure Factor), Figure S1:Peak fit of measured data before application of electric field with the 111 (green) and $1\bar{1}1$ (magenta) peaks shown for reference with the measured data (x), modeled peak fits (red) and residual (black), Figure S2: Peak fit of measured data after application of electric field with the 111(green) and $1\bar{1}1$ (magenta) peaks shown for reference with the measured data (x), modeled peak fits (red) and residual (black). Note the substantial increase in the 111 intensity compared with Figure S1.
