# Supplementary material for: Diffraction Methods for Qualitative and Quantitative Texture Analysis of Ferroelectric Ceramics
Source: Materials (Basel). 2021 Sep 28;14(19):5633. doi: 10.3390/ma14195633 (PMC8510197; doi:10.3390/ma14195633)
Supplement: Supplementary file 1 [file materials-14-05633-s001.zip › materials-1377961-supplementary.pdf]

Article

Diffraction Methods for Qualitative and Quantitative Texture Analysis of Ferroelectric Ceramics †

Chris M. Fancher

Materials Science & Technology Division, Oak Ridge National Laboratory, Oak Ridge, TN 37830, USA; fanchercm@ornl.gov

† This manuscript has been authored by UT-Battelle, LLC under Contract No. DE-AC05-00OR22725 with the U.S. Department of Energy. The United States Government retains and the publisher, by accepting the article for publication, acknowledges that the United States Government retains a non-exclusive, paid-up, irrevocable, world-wide license to publish or reproduce the published form of this manuscript, or allow others to do so, for United States Government purposes. The Department of Energy will provide public access to these results of federally sponsored research in accordance with the DOE Public Access Plan (<http://energy.gov/downloads/doe-public-access-plan>).

Citation: Fancher, C.M. Diffraction Methods for Qualitative and Quantitative Texture Analysis of Ferroelectric Ceramics. *Materials* **2021**, *14*, 5633. https://doi.org/10.3390/ma14195633

Academic Editor: Miguel Algueró

Received: 27 August 2021
Accepted: 17 September 2021
Published: 28 September 2021

Publisher's Note: MDPI stays neutral with regard to jurisdictional claims in published maps and institutional affiliations.

Copyright: © 2021 by the author. Licensee MDPI, Basel, Switzerland. This article is an open access article distributed under the terms and conditions of the Creative Commons Attribution (CC BY) license (http://creativecommons.org/licenses/by/4.0/).

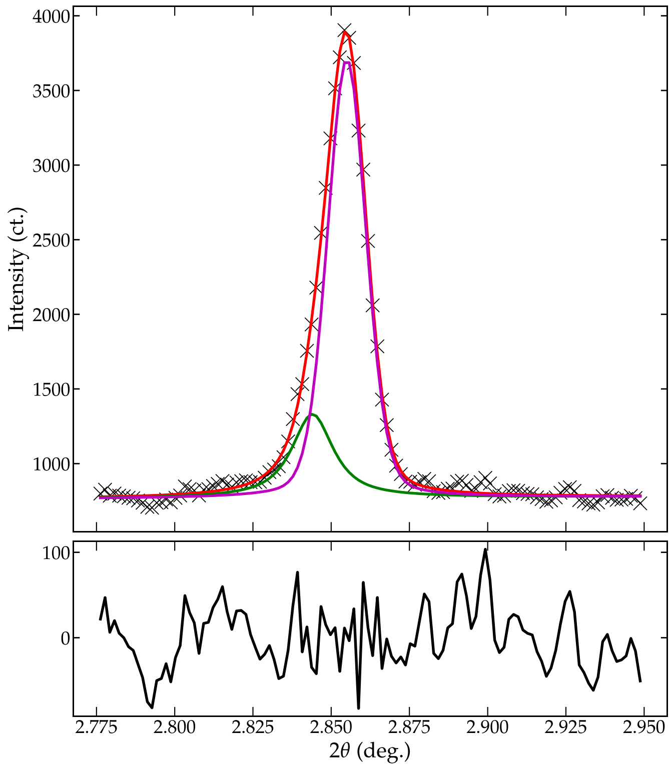

Figure S1. Peak fit of measured data before application of electric field with the 111(green) and $1\bar{1}1$ (magenta) peaks shown for reference with the measured data (x), modeled peak fits (red) and residual (black).

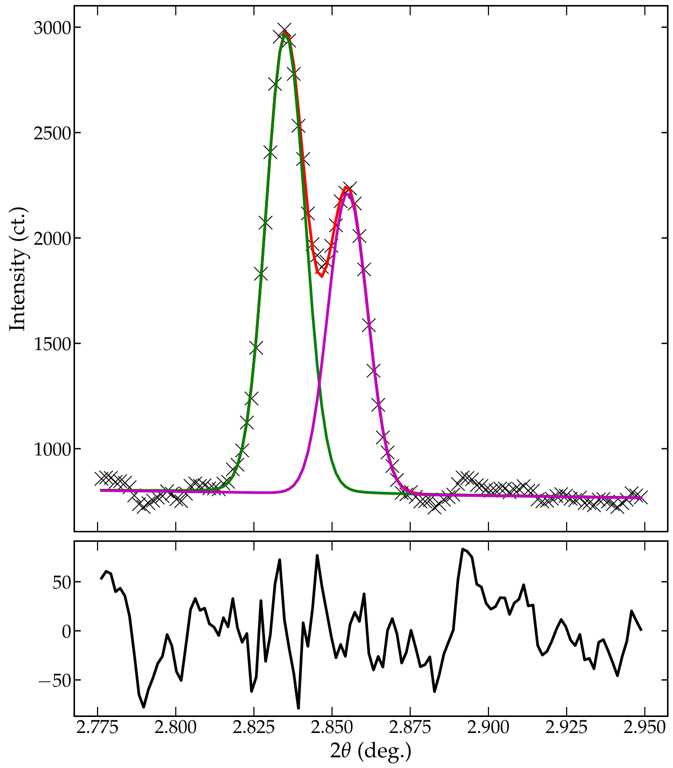

Figure S2. Peak fit of measured data after application of electric field with the 111(green) and 1$\bar{1}$1 (magenta) peaks shown for reference with the measured data (x), modeled peak fits (red) and residual (black). Note the substantial increase in the 111 intensity compared with Figure S1.

Table S1. Summary of the hkl integrated intensity used to determine the p for the templated and p_o randomly oriented metric for an experimental (Powder) and simulated data (Structure Factor).

Reflection	Templated	Powder	Structure Factor
100	117,970(304)	1250(99)	4.67
110	9654(202)	17,261(130)	38.72
111	1608(91)	3966(89)	9.1
200	84,742(509)	6819(96)	14.21
210	15(17)	875(182)	2.97
211	3027(57)	6947(92)	16.12
220	1189(50)	3083(77)	8.29
221/300	3407(66)	1629(80)	4.11
